# Influence of Inlet Splitter Structure on Flow and Heat Transfer Performance in Microchannel Heat Exchangers

**DOI:** 10.3390/mi17020275

**Published:** 2026-02-23

**Authors:** Yuanyuan Xi, Si Chen, Wenchao Tian, Xiong Xiao, Shuaike Li, Feiyang Li, Yifan Wang, Haojie Dang

**Affiliations:** 1 School of Electro-Mechanical Engineering, Xidian University, Xi’an 710071, China; 24041212451@stu.xidian.edu.cn (Y.X.); 24041111090@stu.xidian.edu.cn (F.L.); 24041212624@stu.xidian.edu.cn (Y.W.); d2100710345@outlook.com (H.D.); 2 The Fifth Electronics Research Institute of Ministry of Industry and Information Technology, Guangzhou 511370, China; 3 State Key Laboratory of Electromechanical Integrated Manufacturing of High-Performance Electronic Equipments, Xi’an 710071, China; 4 Pen-Tung Sah Institute of Micro-Nano Science and Technology, Xiamen University, Xiamen 361005, China; xiaoxiong@stu.xmu.edu.cn; 5 State Grid Electric Power Research Institute Co., Ltd. (NARI Group Co., Ltd.), Nanjing 211106, China; lishuaike@sgepri.sgcc.com.cn

**Keywords:** microchannel, inlet and outlet design, PIV, liquid cooling, test platform

## Abstract

Microchannel liquid cooling technology, characterized by high heat-transfer efficiency, represents an effective solution for thermal management in high heat-flux density electronic devices. Existing research has mainly focused on optimizing the structural design of microchannel heat sinks, while neglecting the specific effects of inlet manifold configurations on their heat transfer and flow performance. To obtain more systematic data on microchannel heat transfer performance and internal velocity distribution, this study designed microchannels with single-inlet and triple-inlet configurations. A microchannel cooling performance testing platform was established, and visualization experiments of the internal flow field in straight microchannels were conducted using a particle image velocimetry (PIV) system. The velocity distribution uniformity and heat transfer performance were compared between single-inlet and triple-inlet microchannels with varying channel spacings. The results show that under the same flow conditions, the triple-inlet splitter structure yields a more uniform flow distribution, a lower peak temperature for the heat source chip, and improved heat transfer performance, with its pressure drop reduced to 11.1–26.6% of that of the single-inlet configuration. Furthermore, smaller channel spacings yield improved heat-transfer efficiency in microchannels.

## 1. Introduction

In recent years, the trend toward miniaturization, high density, and high integration in electronic components has placed greater demands on thermal management for electronic devices. High-temperature hotspots in power devices can significantly degrade their operational performance and even cause thermal failure and other reliability problems. The heat flux density generated by next-generation microelectronic devices has reached 1500 W/cm^2^, rendering traditional air cooling ineffective for dissipating such high heat fluxes [1,2].

In 1981, D. B. Tuckerman and colleagues first proposed the concept of microchannel heat sinks. They etched rectangular microchannels onto silicon wafers and conducted cooling experiments using water as the cooling medium. The experimental results demonstrated the exceptional heat dissipation advantages of microchannels: when the chip surface temperature rise was only 71 °C, the chip heat flux density could reach as high as 790 W/cm^2^ [3]. Although the advantages of microchannel liquid cooling are evident, several issues (e.g., high flow resistance induced by the microchannels themselves and abrupt geometric changes, increased system pressure drop, and non-uniform temperature distribution) have consistently hindered its practical application. Therefore, the design of microchannel structures with high heat transfer performance and low system pressure drop remains a key research focus [4].

Researchers have attempted to address issues of high pressure drop and temperature non-uniformity in microchannels through geometric design. Typical microchannel structures include flat-channel designs with grooves [5], corrugated structures [6], and arrayed structures featuring various micro-ribs for turbulence enhancement [7,8]. All these designs enhance the heat transfer capacity by increasing fluid turbulence. Porous structures [9] and biomimetic structures optimized through iterative algorithms [10] can reduce the pressure drop. Regarding heat sink geometry, flat microchannels are the most common structure. The latest research achievement demonstrating significantly superior performance compared to other heat exchangers is the manifold structure proposed by Drummond, K. P. et al. [11]. Following topological layout improvements to the manifold structure and heat source substrate by R. van Erp et al., using deionized water as the working fluid, a pump power of approximately 0.57 W/cm^2^ can dissipate a heat flux density exceeding 1700 W/cm^2^. Compared to conventional flat microchannels, the heat transfer coefficient increased by a factor of 50 [12].

Some studies indicate that even when the cross-sectional geometry and inlet/outlet velocities of microchannels are identical, significant differences in pressure drop and heat dissipation capacity can occur if the inlet cavity topology varies [13]. To enhance flow diversion efficiency and improve flow field uniformity, methods such as modifying the inlet cavity shape, adjusting the inlet position, and installing flow-guiding structures can be employed. Dahiya A. et al. [14] employed numerical simulation to compare the heat transfer coefficients and Nusselt numbers of three different microchannel designs with straight inlet cavities: rectangular, U-shaped, and trapezoidal. Results indicate that within the Reynolds number range of 342 to 857, the trapezoidal inlet cavity exhibits the highest heat transfer coefficient, followed by the U-shaped inlet cavity, while the rectangular inlet cavity demonstrates the lowest coefficient. The trapezoidal heat transfer coefficient is approximately 9% to 10% higher than that of the rectangular cavity.

The spatial relationship between the inlet direction and the flow channel direction also significantly impacts the heat transfer performance. Reiyu Chein et al. [15] employed numerical simulation methods to study the effects of the inlet position on flow field distribution and temperature uniformity in flat microchannel heat sinks with five distinct inlet configurations. The results indicate that, under identical pressure drops, the U-type and V-type structures with vertical inlet/outlet configurations exhibit more uniform velocity distributions and superior overall heat transfer performance compared to the three configurations featuring horizontal fluid entry into the microchannel.

Modifying the number of inlets can also enhance heat transfer performance. Xia Y. et al. [16] conducted numerical simulations on microchannels with different inlet and outlet configurations. The results indicated that the four-inlet configuration exhibited the lowest dimensionless maximum temperature, demonstrating its superior heat transfer performance. Incorporating flow divider structures within the inlet cavity is another effective method to enhance flow distribution uniformity. Lian T. et al. [17] integrated microchannel structures within a 2.5D packaging architecture, incorporating diverging flow structures at the inlet and outlet sections of straight microchannels. Deionized water was circulated through the channels to directly cool high-power gallium nitride (GaN) high electron mobility transistors (HEMTs). Lian T et al. evaluated the heat transfer performance of this structure through experiments and simulations. The results indicate that at a flow rate of 3 mL/min, the thermal flux density at the gate of the GaN HEMT device reached 32 kW/cm^2^, while the average thermal flux density at the source was 5 kW/cm^2^. The maximum surface temperature of the device could be reduced to 93.8 °C.

The existing flow characteristic evaluation methods assess in-channel flow solely by measuring the inlet velocity. Most studies [18,19] derive the average flow velocity from inlet flow rate measurements, neglecting the specific impact of flow distribution uniformity. This approach fails to capture the actual velocity distributions and flow resistance trends within microchannels, making it difficult to accurately elucidate their heat dissipation mechanisms. In fact, the flow velocity and velocity uniformity of the coolant within the channel are among the primary factors influencing the flow resistance and pressure drop in microchannels. Enhancing the heat dissipation capability of microchannels through improvements in inlet cavity topology is achieved precisely by altering the velocity distribution within the channel.

To address the aforementioned issues, this study designed two microchannel inlet diverter structures—single-inlet and triple-inlet—and constructed a cooling test circuit for evaluating microchannel heat dissipation performance. Experiments utilizing PIV-based velocity measurements captured the internal flow velocity distributions within the straight microchannels of both cooling circuits. The study compared the effects of inlet diverging structures and channel spacing on the heat transfer performance and flow characteristics, including the flow field velocity distribution uniformity, junction temperature of the heat source chip, and system pressure drop.

## 2. Materials and Methods

### 2.1. Experimental Design

#### 2.1.1. Microchannel Sample Design and Dimensions

**Figure 1:**
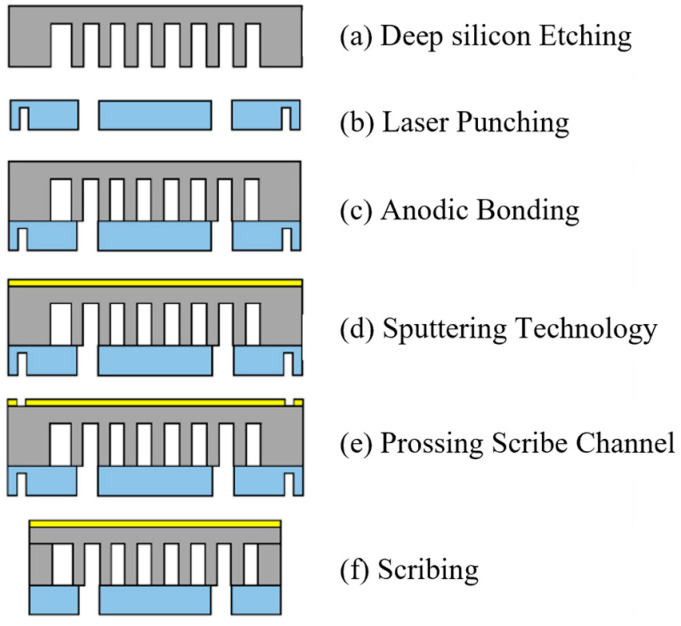
Microfluidic test sample fabrication process flow.

The internal channels of the microfluidic samples were fabricated by deep silicon etching, and the samples were prepared via silicon-glass anodic bonding. This design enables direct visualization of the flow field inside the channels through the glass surface. The specific preparation procedure is outlined as follows (Figure 1a–f):

(a) Prepare several silicon wafers and glass substrates for wafer fabrication. Both materials have a thickness of 0.5 mm. Perform deep silicon etching through the following steps: photoresist coating, pre-bake, exposure, development, microscopic inspection, hardening, and oxygen plasma residue removal. Complete the photolithography process to etch trench patterns onto the silicon wafers.
(b) Use laser drilling to create 1 mm-diameter through-holes in the glass plate; these holes serve as the working fluid inlet/outlet ports.
(c) After inorganic cleaning of the silicon wafers and glass substrates with concentrated sulfuric acid and hydrogen peroxide, achieve silicon-glass anodic bonding by subjecting the assembly to pressure treatment at specific temperatures and voltages.
(d) Deposit a Ti/Au layer on the silicon wafer surface via sputtering to facilitate subsequent heat source chip bonding.
(e) Apply a dry film to the glass surface for protection; simultaneously, create dicing grooves on the silicon wafer surface via photolithography. Subsequently, use an Au/Ti etchant to remove the metal in the dicing groove areas.
(f) Generate the final microchannel sample after sectioning.

**Figure 2:**
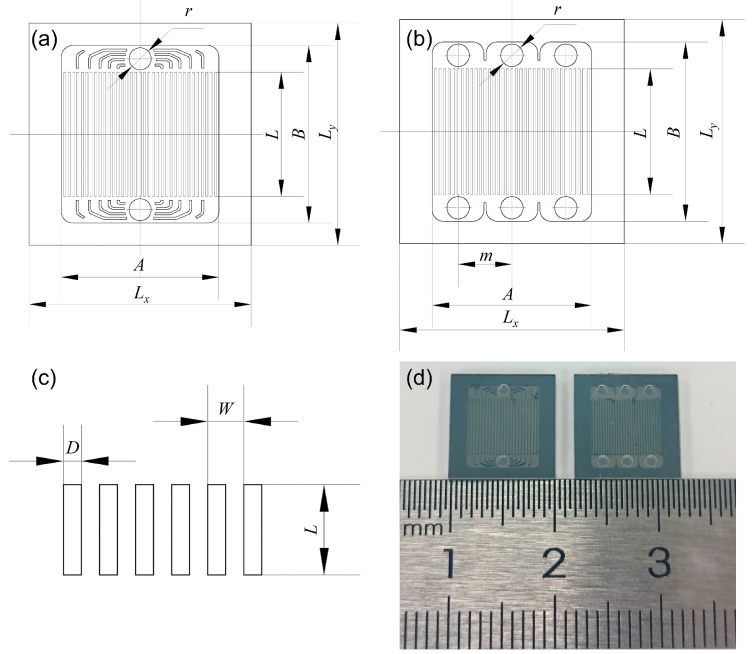
Straight microchannel sample diagram.

As shown in Figure 2a depicts the dimensional schematic of a single-inlet microchannel structure. The overall dimensions of the microchannel sample are L_x_ = 10 mm and L_y_ = 10 mm. The inlet and outlet diameters r = 1 mm. The fluid region measures A = 8 mm in length and B = 7 mm in width, with a channel etching depth of H_ch_ = 0.25 mm. Multiple channels are distributed within a rectangular region (length A, width L), with chamfered flow-splitting structures incorporated at the inlet and outlet. Figure 2b shows the dimensional schematic of a triple-inlet microchannel structure, where the inlet spacing m = 2.3 mm, and micro-ribs separate each inlet.

The dimensions of the straight-type channel structure are shown in Figure 2c. Three channel spacings were designed for each channel type, with each spacing featuring both single-inlet (Single) and triple-inlet (Triple) configurations. Specific geometric parameters are listed in Table 1. The sample designation in Table 1 follows the naming convention “inlet configuration-channel spacing,” e.g., “Triple-0.25” denotes a straight microchannel with triple inlets and a channel spacing of 0.25 mm. Figure 2d shows physical images of microchannel samples with a channel spacing of 0.20 mm and different inlet configurations: the left image depicts a single inlet, while the right image shows a triple inlet.

#### 2.1.2. Heat Source Chip

**Figure 3:**
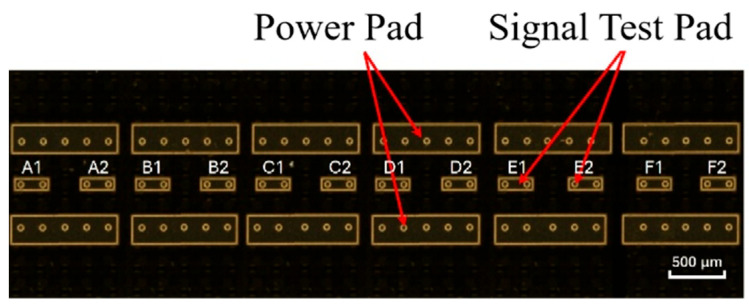
Enlarged view of the actual thermal test chip.

The thermal test chip serves as a simulated heat source, integrating a heating resistor and multiple thermistors to achieve both heat generation and temperature measurement. Its temperature measurement principle operates as follows: when the resistor is energized, the chip generates heat. Under constant current drive, the forward voltage of the thermistor changes linearly with the temperature. By measuring the voltage across the thermistor terminals, the actual temperature of the heat source chip can be determined. As shown in Figure 3, the thermal test chip consists of six independent temperature measurement units. Each unit includes two heating pads (upper and lower) and two temperature measurement pads (middle). The units are numbered from left to right as A, B, C, D, E, and F. The twelve temperature measurement pads are numbered from left to right as A1, A2, B1, B2 … F1, and F2. The thermal test chip is flip-chip mounted onto a PCB substrate pre-fabricated with load terminals and feedback signal test points to complete feedback signal measurement. After the chip temperature stabilizes, a high-precision multimeter (Model 2000, Keithley Instruments, Inc., Cleveland, OH, USA) is used to measure the chip’s feedback signal.

#### 2.1.3. Microchannel Test Fixture

**Figure 4:**
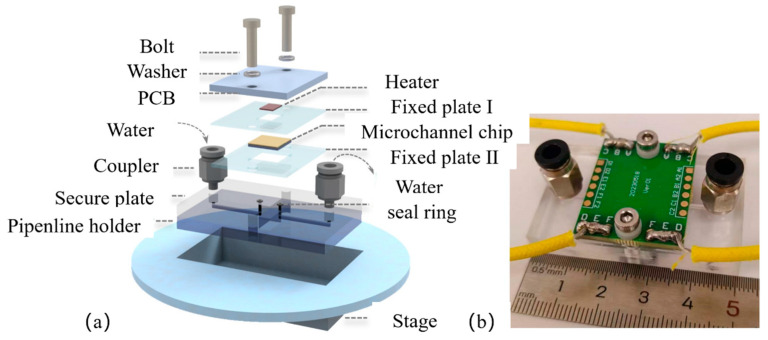
Microfluidic channel fixture: (**a**) exploded view of microfluidic channel test fixture model and (**b**) actual image of microfluidic channel test fixture.

The exploded view of the microchannel test fixture model is shown in Figure 4a. Its main body consists of two PMMA (polymethyl methacrylate) plates—a secure plate and a pipeline holder—each engraved with working fluid flow channels and thermally bonded together. First, photolithography and dry etching are used to etch the flow channel structure onto the pipeline holder with an etch depth of Hp = 1 mm. Threaded through-holes are machined at the inlet and outlet positions on the secure plate to accommodate couplers for external tubing connections. A rectangular through-hole is machined at the corresponding central position of the microchannel chip to reserve a field of view, preventing subsequent microscope imaging from being affected by glass refraction or substrate impurities. Sealing ring positioning grooves are provided at the inlet and outlet of the microchannel sample to accommodate rubber washers, preventing fluid leakage due to inadequate sealing. Fixed plate II is a PMMA plate measuring 30 mm × 25 mm in length and width, with a thickness of 1 mm. It features a 10 mm × 10 mm square through-hole for positioning the microchannel chip. Fixed plate I is a PMMA plate measuring 30 mm × 25 mm in length and width, with a thickness of 0.5 mm. It features an 8 mm × 6 mm rectangular through-hole for clamping and securing the microchannel chip. Assemble the secure plate, pipeline holder, PCB, fixed plate I, fixed plate II, and microchannel chip, using bolts. Place a washer beneath the clamp bolt to prevent damage from loosening or excessive preload. High thermal conductivity grease fills the groove in the microchannel cover plate to facilitate heat transfer between the heat source chip and the microchannel. To secure the test section and ensure a clear, stable field of view under the microscope, a microscope stage was fabricated using 3D printing technology. Placing the fixture into the square hole prevents minor displacement of the microchannel fixture during experiments. Figure 4b shows the physical image of the microchannel fixture.

**Figure 5:**
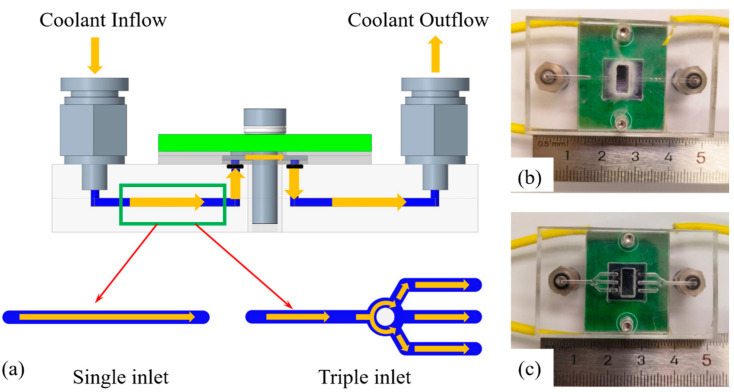
Schematic diagram of drainage structures: (**a**) schematic model of drainage structure, (**b**) actual image of single-inlet drainage structure and (**c**) actual image of triple-inlet drainage structure.

Figure 5a shows schematic diagrams of the single-inlet and triple-inlet fixture drainage structures, both with channel widths of 1 mm. The fluid from the triple-inlet fixture’s flow channel is diverted by an intermediate cylindrical baffle, entering the upper, middle, and lower channels, respectively, before entering the respective inlet ports of the triple-inlet microfluidic channel. The upper and lower bifurcated channels are arranged at a 60° angle to the middle channel. Figure 5b,c show the physical images of the single-inlet flow structure and the triple-inlet flow structure, respectively.

### 2.2. Test Setup

**Figure 6:**
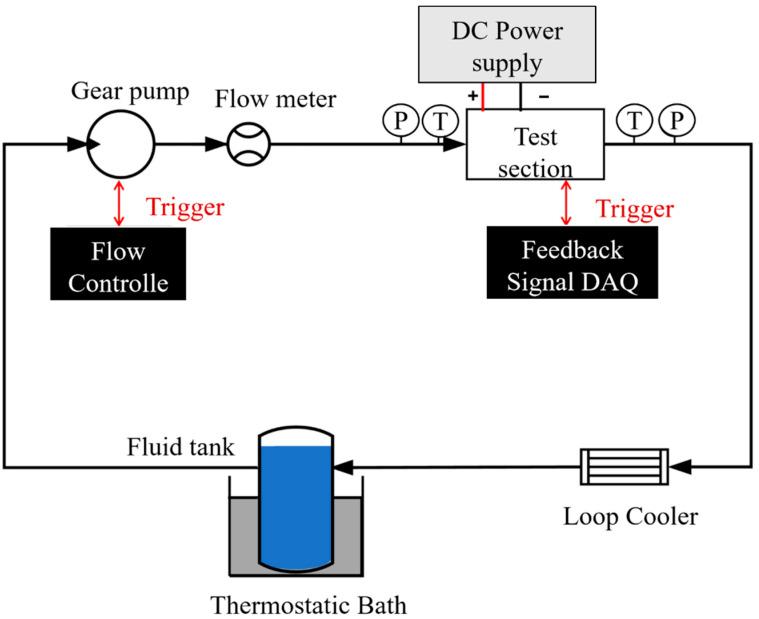
Schematic diagram of heat exchange performance test circuit.

As shown in Figure 6, the microchannel heat transfer performance test circuit consists of a fluid tank, a thermostatic bath, a gear pump (Model GAF-T23, Micropump, Vancouver, WA, USA), a flowmeter (Model 1111/DN0.7, GICAR, Xi’an, Shaanxi, China), a pressure transmitter (Model 3510B0010A0LER00, Gems Sensors & Controls, Plainville, CT, USA), a test section, and a loop cooler.

To keep the coolant in the fluid tank at a constant temperature, the tank is placed in a thermostatic bath to minimize the experimental errors caused by the temperature fluctuations of the coolant. Each component is connected via polyurethane (PU) tubing with an inner diameter of 2.5 mm. Deionized water is used as the coolant. The coolant is driven by a gear pump, flowing from the fluid tank through the PU tubing into the gear pump inlet. The gear pump outlet connects to a flowmeter. After passing through the flowmeter, the coolant flows through PU tubing connected via quick-connect fittings to a three-way quick-connect fitting. At the outlet perpendicular to the flow direction, an external pressure transmitter is connected to measure the inlet pressure. After passing through the inlet pressure transmitter, the coolant enters another three-way quick-connect fitting. A thermocouple wire is inserted into the outlet perpendicular to the flow direction to measure the inlet temperature. After passing the inlet thermocouple, the coolant flows through the microchannel test section. It then passes through a thermocouple measuring the outlet temperature and a pressure transmitter measuring the outlet pressure. The heated working fluid flows into the loop cooler for cooling before returning to the reservoir, completing the test circuit.

To prevent fluid leakage and ensure the integrity of the entire circuit, we fill the threaded connections with PTFE tape. While ensuring full contact between the thermocouple measurement point and the liquid, we apply sealant to fill the connection between the thermocouple and the quick-connect tee fitting.

**Figure 7:**
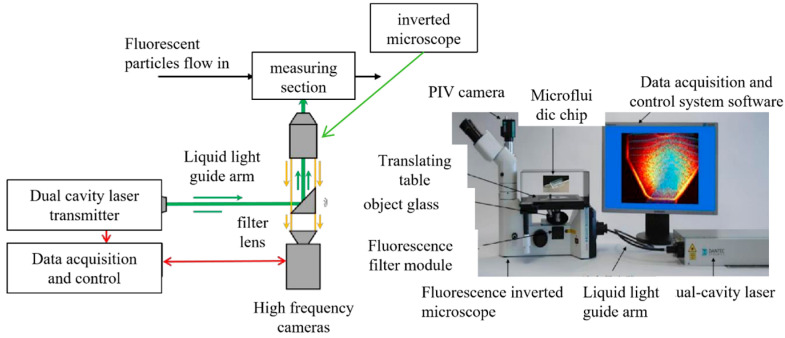
Schematic diagram of the PIV system.

The schematic diagram of the PIV system’s velocity measurement principle is shown in Figure 7. Fluid velocity is calculated by measuring particle displacement over short time intervals: first, nanoscale, highly reflective fluorescent particles are dispersed in the fluid medium, and then the working fluid containing these particles is introduced into the flow channel. Two high-frequency laser beams with an extremely short time interval are continuously directed at the fluid region to obtain multiple sets of results, each comprising two photographs captured at different exposure times. Finally, based on the particle displacement in the two photographs and the laser exposure interval, the velocity distribution across the entire flow field is determined through cross-correlation calculations.

The PIV system (Dynamic Studio 2D2C MicroPIV, Dantec Dynamics, Skovlunde, Denmark) used in this study is configured with components including a dual-cavity laser emitter, liquid light guide arm, fluorescence inverted microscope, PIV camera, synchronous controller, displacement stage, and data acquisition computer. The dual-cavity laser emitter generates two laser beams at short intervals to illuminate the flow field. The microscope, paired with the PIV camera—a high-speed camera capable of up to 40 fps at 5.5 megapixel resolution—captures images of microscopic flow channels illuminated by laser flashes. The displacement stage controls small-displacement movements of the sample. The synchronization controller facilitates signal transmission between all components.

Prasad et al. [20] conducted research to reduce measurement errors in PIV systems. Their findings indicate that measurement errors are minimized when the diameter of particles projected onto a high-speed camera is approximately twice the pixel diameter. Based on this, this study observed the distribution of fluorescent particles at different concentration ratios. Adding fluorescent particles (Invitrogen Nile Red-F8819 GB/T 7714-2015) with a particle size of 1 μm and a volume fraction of 0.2% to deionized water enables relatively accurate real-time capture of particle images.

**Figure 8:**
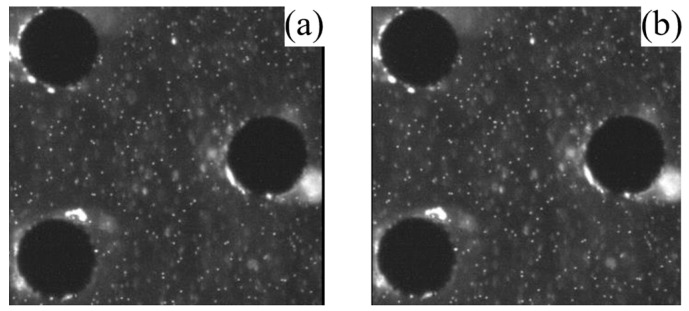
Fluorescent particle images at the same location within the flow channel. (**a**) First frame particle image. (**b**) Second frame particle image.

An important parameter of the PIV system is the interframe time, defined as the interval between exposures of the two lasers; a shorter interframe time corresponds to a shorter interval between the two frames. When the flow velocity is high, the interframe time should be reduced to obtain two frames with suitable relative displacement. For optimal flow field calculations, a single particle should move 5–8 pixels across the two exposed frames, as shown in Figure 8.

To compare the impact of flow rate on microchannel heat dissipation performance, the inlet coolant flow rates were set to 12, 16, 24, 35, 48, and 60 mL/min. The inlet coolant temperature was 27 °C, the test chip power was 3.3 W, and the heat flux density was 25 W/cm^2^.

Heat flux density calculation formula for the heat source chip:

(1) $$q_{w}=\frac{P}{A_{chip}}$$

In the formula, *P* is the power loaded onto the heat source chip and *A_chip_* is the chip surface area, calculated as 0.132 cm^2^.

The calibration method for the flow meter involves combining the volume of liquid delivered per minute with the measuring cylinder, ultimately achieving an error range of ±0.8%. The calibration of the thermal test chip utilizes a T3ster testing instrument to verify the temperature–voltage curve of the thermal test chip. The sensitivity of the temperature-sensing diode in this chip to temperature changes is 1.85 mV/°C. The calibration process for the digital thermocouple temperature gauge involves attaching the thermocouple measurement point to a heating platform. At preset 10 °C temperature gradients between 30 °C and 100 °C, the gauge’s displayed parameters are measured, yielding a final average error of ±0.67%. Table 2 presents the experimental uncertainty table.

Based on flow velocity variations within the channel, the PIV system’s interframe time interval Dt was selected within the range of 2 μs–50 μs.

### 2.3. PIV Field of View Selection

**Figure 9:**
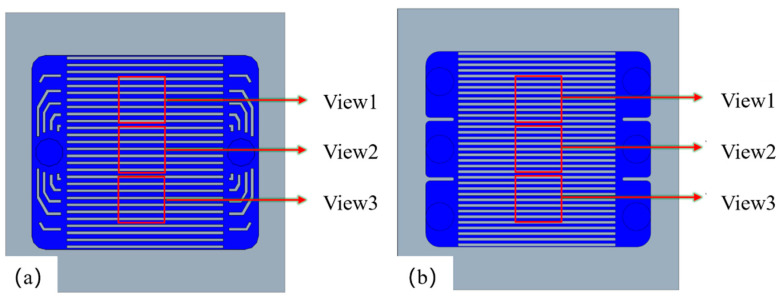
Schematic of PIV system field of view. (**a**) Single-inlet microchannel. (**b**) Triple-inlet microchannel.

To obtain the velocity distribution within the flow channel after the fluid has fully developed, particle image positions should be captured at the center of the channel. The PIV field of view acquisition regions for the two inlet configurations of the microchannel are shown in Figure 9. Each individual field of view spans a rectangular area measuring 1.5 mm × 1.5 mm, designated as View1, View2, and View3. Figure 9a illustrates the single-inlet field of view range. The field of view for the triple-inlet configuration is identical to that of the single-inlet configuration. Figure 9b illustrates the field of view for the triple-inlet microchannel.

### 2.4. Numerical Simulation Methods

#### 2.4.1. Description of the Mathematical Model

Using deionized water as the coolant, the following physical assumptions were made to simplify the numerical model:

(1) The coolant is an incompressible Newtonian fluid;
(2) Gravity and other volumetric forces are neglected;
(3) Thermal radiation is ignored, considering only thermal conduction and convection;
(4) The effects of viscous dissipation are neglected;
(5) The thermal conductivity and dynamic viscosity of the coolant vary with temperature. Linear interpolation is employed to input the temperature-dependent thermal conductivity and dynamic viscosity into the Fluent (Ansys 19.2) software [21].

For convective heat transfer problems involving incompressible, three-dimensional fluids without internal heat sources, the governing equations are derived based on the three classical principles of mass conservation, momentum conservation, and energy conservation [22].

The mass conservation equation, also known as the continuity equation, is shown in Equation (2):

(2) $$\frac{\partial u}{\partial x}+\frac{\partial v}{\partial y}+\frac{\partial w}{\partial z}=0$$

The momentum conservation equation, namely the Navier–Stokes equation, is expressed as shown in Equations (3) to (5):

(3) $$\rho_{f}\left(u\frac{\partial u}{\partial x}+v\frac{\partial u}{\partial y}+w\frac{\partial u}{\partial z}\right)=\mu_{f}\left(\frac{\partial^{2}u}{\partial x^{2}}+\frac{\partial^{2}u}{\partial y^{2}}+\frac{\partial^{2}u}{\partial z^{2}}\right)-\frac{\partial p}{\partial x}$$

(4) $$\rho_{f}\left(u\frac{\partial v}{\partial x}+v\frac{\partial v}{\partial y}+w\frac{\partial v}{\partial z}\right)=\mu_{f}\left(\frac{\partial^{2}v}{\partial x^{2}}+\frac{\partial^{2}v}{\partial y^{2}}+\frac{\partial^{2}v}{\partial z^{2}}\right)-\frac{\partial p}{\partial y}$$

(5) $$\rho_{f}\left(u\frac{\partial w}{\partial x}+v\frac{\partial w}{\partial y}+w\frac{\partial w}{\partial z}\right)=\mu_{f}\left(\frac{\partial^{2}w}{\partial x^{2}}+\frac{\partial^{2}w}{\partial y^{2}}+\frac{\partial^{2}w}{\partial z^{2}}\right)-\frac{\partial p}{\partial z}$$

The energy conservation equation, as shown in Equation (6):

(6) $$u\frac{\partial t}{\partial x}+v\frac{\partial t}{\partial y}+w\frac{\partial t}{\partial z}=\frac{\lambda_{f}}{\rho_{f}Cp}\left(\frac{\partial^{2}t}{\partial x^{2}}+\frac{\partial^{2}t}{\partial y^{2}}+\frac{\partial^{2}t}{\partial w^{2}}\right)$$

In the equation: *u*, *v* and *w* represent the velocity components in the *x*, *y* and *z* directions, respectively. *P* denotes the pressure of the coolant, and *μ_f_* denotes the dynamic viscosity of the coolant. *t_f_* denotes the temperature of the coolant, $\lambda_{f}$ denotes the thermal conductivity of the coolant, and *C_p_* denotes the specific heat capacity of the coolant.

The above equations apply to both laminar and turbulent flow conditions for incompressible viscous fluids.

#### 2.4.2. Model Development

**Figure 10:**
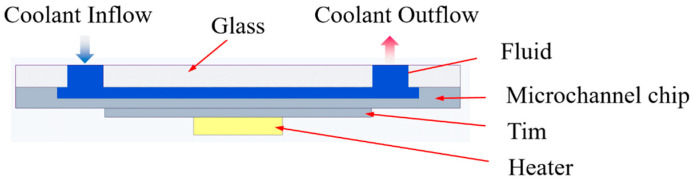
Schematic diagram of the simulation model.

Figure 10 shows a schematic diagram of the microchannel simulation model. The fin width of the straight-type fin channel is set to 100 μm. The arrows indicating the coolant inlet and outlet represent the flow direction of the coolant. The inlet and outlet diameters are 1 mm. The model was constructed proportionally, based on the channel parameters shown in Figure 2.

The microchannel substrate employs silicon material with high thermal conductivity, enabling heat from the heat source chip to be conducted through thermal interface material and the microchannel substrate into the fluid domain. To ensure uniform velocity distribution within the channels and to facilitate fluid entry into edge channels, a guide plate design is implemented. This reduces flow separation in the inlet section, ensuring fair comparison. The triple-inlet configuration achieves uniform fluid distribution by adding bifurcated channels (arranged at 60° angles) to the single-inlet channel. Comparing the two structures directly reflects the impact of the inlet bifurcation design. Visualization experiments were conducted to observe the internal flow field, necessitating the use of transparent polymethyl methacrylate (PMMA) for the encapsulation cover. The dimensional parameters of the three-dimensional models for each component are shown in Table 3.

#### 2.4.3. Boundary Conditions

Based on calculations, the laminar flow model was selected as the model for this simulation [23]. In the boundary conditions setup, the inlet was configured as a “velocity inlet” with a temperature of 26.85 °C. The outlet is set to “pressure outlet”, with a relative pressure of 0 MPa. Standard wall conditions were applied to the surfaces, with an external temperature set to 22 °C. Under General, the solution type was set to a steady-state with gravity ignored. In the solution scheme, the coupled mode was selected, the pseudo-transient option was enabled, and all other settings remained unchanged. During initialization, 15 iterations were specified. During the solution process, 150 iterations were set. Convergence is deemed as being achieved when the residual of the energy equation falls below 10^−6^ and the residuals of other equations drop below 10^−3^. To more realistically simulate the chip’s operating environment, the loading conditions were determined based on the design criterion of maintaining the chip’s safe temperature below 75 °C. The final thermal flux density for the chip was set at 25 W/cm^2^. The inlet volumetric flow rates matched the test conditions: 12, 16, 24, 35, 48, and 60 mL/min, with corresponding inlet velocities of 0.25, 0.34, 0.51, 0.74, 1.02, and 1.27 m/s.

#### 2.4.4. Mesh Partitioning and Independence Verification

A tetrahedral mesh partitioning method is employed for mesh generation, followed by mesh independence verification to determine whether the mesh quality meets the computational accuracy requirements. The grid independence verification process involves progressively increasing the mesh density from low to high levels. Simulation results for key parameters at different densities are compared to determine the relative error. The primary simulation outputs extracted are the maximum surface temperature (*T*_max_) and inlet–outlet pressure drop (Δ*P*). The relative error *e* is calculated according to Equation (7) [24]:

(7) $$e=\frac{\left|E_{i}-E_{i+1}\right|}{E_{i}}\times 100$$

In the formula, *E_i_* and *E_i+_*_1_ are the primary parameter values of two adjacent grid densities.

**Figure 11:**
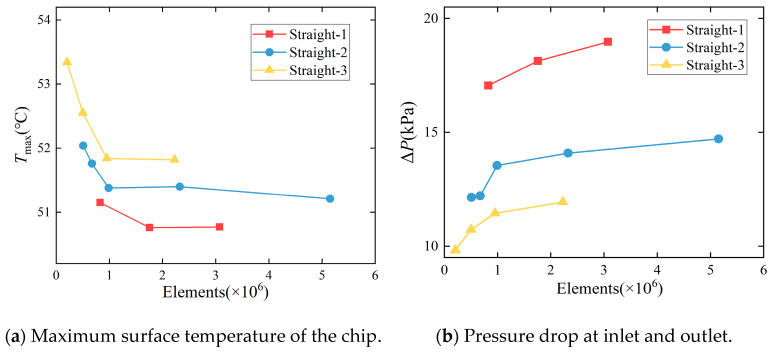
Comparison of grid-independent verification for straight microchannel patterns.

Figure 11 shows a comparison of the maximum temperature (*T*_max_) and inlet-outlet pressure drop (Δ*P*) for a straight microchannel chip.

When the relative error of the maximum temperature on the chip surface is less than 1% and the relative error of the inlet–outlet pressure drop is less than 5%, the final number of meshes for each microchannel structure is determined as shown in the table below. The table indicates that compared to the previous level of meshes, the relative errors of both and the final selected number of meshes meet the error criteria, demonstrating that mesh independence has been achieved. Table 4 presents the relative error statistics.

## 3. Results and Discussion

### 3.1. Analysis of Flow Field Distribution Results

#### 3.1.1. Comparison of Flow Velocity Distribution

**Figure 12:**
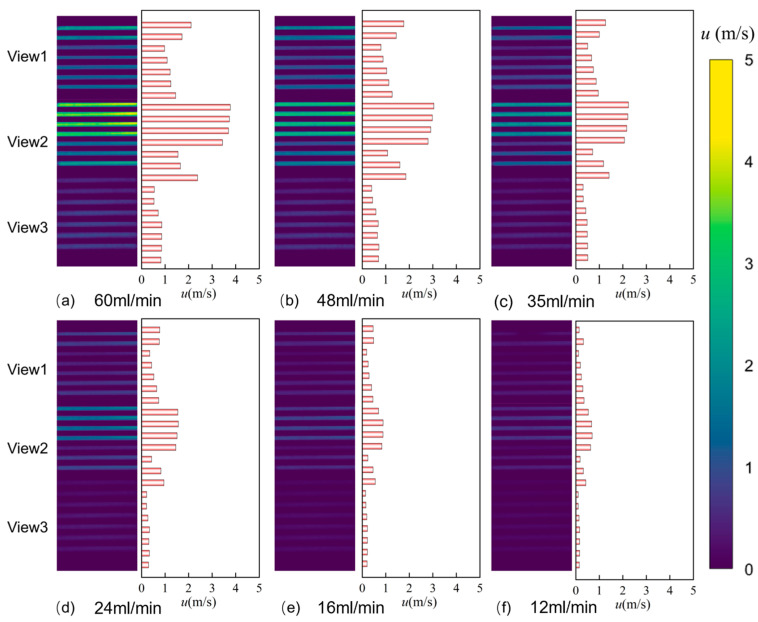
PIV velocity distribution maps of the Single-0.20 microchannel at different flow rates: (**a**) 60 mL/min, (**b**) 48 mL/min, (**c**) 35 mL/min, (**d**) 24 mL/min, (**e**) 16 mL/min, and (**f**) 12 mL/min.

Figure 12 shows the PIV velocity distribution contour plots for the Single-0.20 microchannel under different flow conditions. As shown in Figure 12a, at a volumetric flow rate of 60 mL/min, the flow velocity in the Single-0.20 microchannel presents a unimodal distribution, with a higher velocity in the central channel and lower velocities in the side channels. The maximum velocity of 3.77 m/s occurs within the View2 field of view, while the minimum velocity of 0.53 m/s is observed within the View3 field of view. Comparing the locations of maximum velocities across different flow rates reveals consistent positioning. Furthermore, the maximum velocity decreases as the flow rate diminishes; at 12 mL/min, the maximum velocity drops to 0.69 m/s.

**Figure 13:**
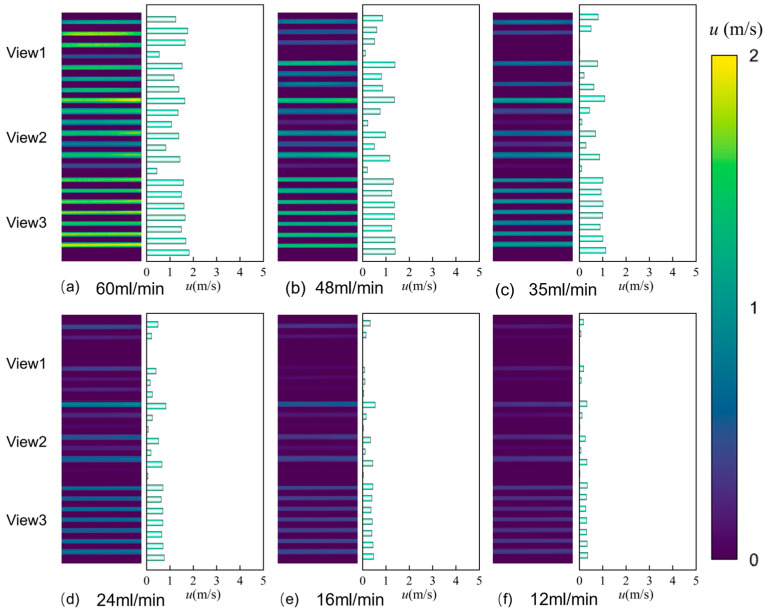
PIV velocity distribution maps of the Triple-0.20 microchannel at different flow rates: (**a**) 60 mL/min, (**b**) 48 mL/min, (**c**) 35 mL/min, (**d**) 24 mL/min, (**e**) 16 mL/min, and (**f**) 12 mL/min.

As shown in Figure 13a, compared to the single-inlet straight microchannel, the flow velocity in the triple-inlet channel did not exhibit a pronounced single-peak trend at a flow rate of 60 mL/min. Instead, significant flow velocities were observed across both fields of view, with minimal velocity fluctuations. Comparing the maximum flow velocities in each channel across different flow rates in Figure 13a–f, the maximum velocity decreased from 1.82 m/s to 0.36 m/s as the flow rate decreased from 60 mL/min to 12 mL/min. The maximum flow velocity diminishes as the flow rate decreases.

Comparing the PIV flow visualization results of the single-inlet and triple-inlet configurations reveals that the inlet flow rate significantly affects the flow velocity in both layouts, though their flow velocity response patterns exhibit distinct differences. In the single-channel configuration, when flow rate decreases from 60 mL/min to 12 mL/min, velocities across all observation fields (Views 1–3) show a sharp decline. In contrast, the three-channel configuration exhibited a significantly lower overall velocity level, with maximum speeds at 60 mL/min reaching only 1.5–2.0 m/s. Additionally, the color distribution across all fields of view was more uniform, and histograms showed velocities that were consistently concentrated within the 0–2 m/s range. This indicates that the multi-channel diversion structure effectively suppresses the formation of localized high-velocity zones, promoting a more spatially uniform flow. The spatial distribution characteristics of flow in the two channel configurations further reveal the regulatory mechanism of channel number on flow uniformity.

In single-channel flow, View 2 consistently represents the high-velocity core region, while Views 1 and 3 maintain low-velocity states. This vertical velocity stratification reflects the preferential aggregation effect of the fluid in the single-inlet channel. In stark contrast, the velocity color differences across fields of view are significantly reduced in the three-channel configuration. The high-velocity zone becomes more dispersed across different fields of view, indicating that the bifurcation structure weakens the localized velocity concentration effect by increasing lateral diffusion pathways. This contrast not only demonstrates the direct influence of the channel configuration on flow dynamics but also provides intuitive experimental evidence for optimizing velocity uniformity in microfluidic devices.

PIV velocity distribution plots for the Single-0.25, Single-0.30, Triple-0.25, and Triple-0.30 microchannels under different flow rates are shown in Appendix A.

At a volume flow rate of 60 mL/min, the regions of maximum flow velocity for both the Single-0.25 and Single-0.30 channel spacings were located within View2, with maximum velocities of 2.39 m/s and 1.07 m/s, respectively. As flow decreased to 12 mL/min, velocities dropped to 0.37 m/s and 0.14 m/s, respectively. Similar to Single-0.20, Single-0.25 exhibited a pronounced unimodal velocity trend, with higher velocities in View2 than in View1. In the Single-0.30 configuration, the differences in maximum flow velocity across fields of view were not significant. At a volume flow rate of 60 mL/min, the maximum flow velocities in the Triple-0.25 and Triple-0.30 microchannels were both located within View2, at 0.98 m/s and 1.21 m/s, respectively. As flow rate decreased to 12 mL/min, maximum velocities dropped to 0.17 m/s and 0.22 m/s, respectively. Comparing maximum velocities across different channel spacings at the same flow rate revealed that for the triple-inlet microchannel, the maximum velocity first decreased and then increased with increasing channel spacing.

**Figure 14:**
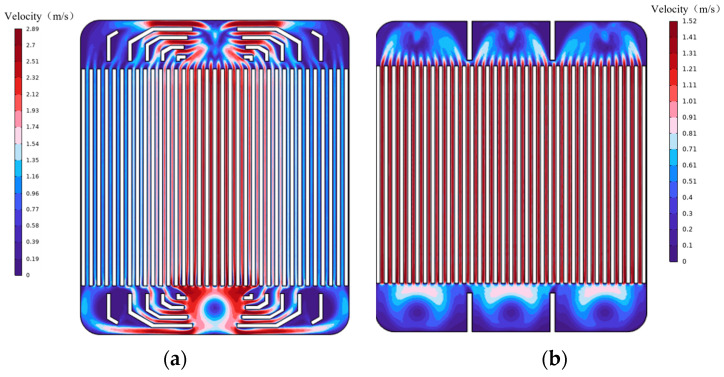
Numerical simulation velocity distribution contour plots for microchannels with a flow channel spacing of 0.20 mm at a flow rate of 60 mL/min: (**a**) single-inlet microchannel distribution plot and (**b**) three-inlet microchannel distribution plot.

**Figure 15:**
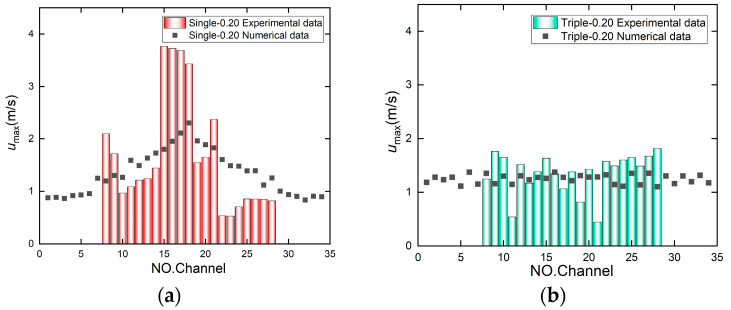
Comparison of experimental and simulated velocity distributions for a microchannel with 0.20 mm inter-channel spacing at a flow rate of 60 mL/min. (**a**) Single-inlet velocity distribution comparison. (**b**) Triple-inlet velocity distribution comparison.

At a flow rate of 60 mL/min, the numerical simulation velocity distribution contour map for the microchannel with a channel spacing of 0.20 mm shows a higher flow velocity in the central channel. Figure 14 shows the velocity distribution profile from the numerical simulation. Figure 15 presents the comparison between the experimental and simulation results. The simulation for the single-inlet configuration shows a “single-peak” trend with higher flow velocity in the middle channel and lower velocities in the side channels. In contrast, the PIV experimental results within View2 correspond to the middle channel, exhibiting a higher flow velocity, which is consistent with the single-peak trend (higher in the middle, lower on both sides) depicted in the simulation results (Figure 14). In contrast, the experimental flow velocity in the three-channel configuration consistently remained within a narrow range of 0–2 m/s with minimal fluctuations, exhibiting higher agreement with the numerical simulation results. This discrepancy indicates that the three-channel configuration not only effectively suppresses the formation of localized high-velocity zones but also enhances the flow stability. This consistency between experimental measurements and numerical predictions further validates the role of multi-channel flow diversion in regulating flow velocity uniformity.

#### 3.1.2. Comparison of Diversion Effects

**Figure 16:**
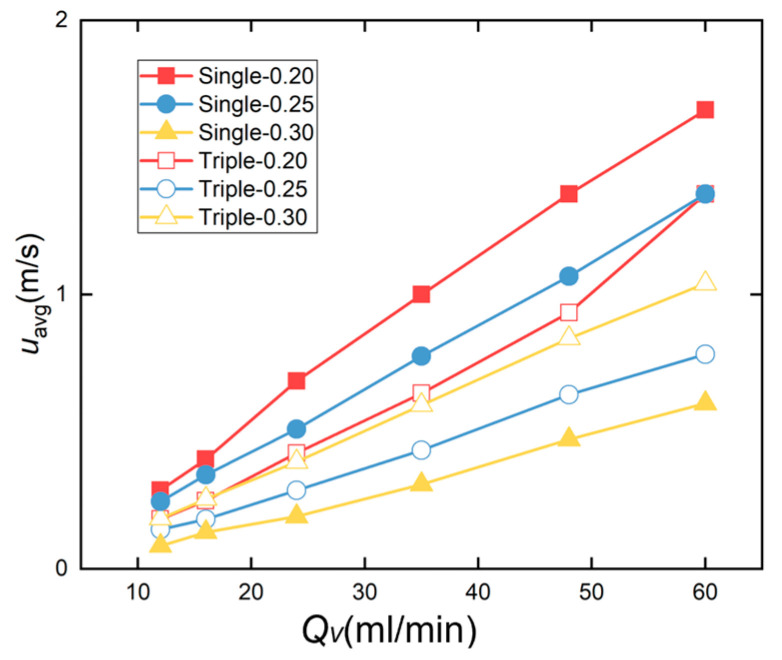
Variation in average flow velocity *u*_avg_ with flow rate *Q_v_* in a straight microchannel.

Figure 16 shows the variation curve of the average flow velocity *u*_avg_ at the minimum cross-section, with respect to flow rate *Q_v_*. The figure indicates that, compared to a single-inlet straight microchannel at a flow rate of 60 mL/min, the average flow velocity decreased from 2.14 m/s to 0.61 m/s as the channel spacing increased from 0.20 mm to 0.30 mm. This trend was also observed under other flow rate conditions.

Compared to the other two straight microchannels, the Triple-0.20 exhibits a higher average flow velocity than the other two channel spacings. However, as the flow rate decreases below 35 mL/min, the difference in average flow velocity becomes negligible, with all microchannels showing similar average flow rates. Overall, the Triple-0.20 channel with 0.20 mm spacing exhibited the highest average flow velocity, followed by the Triple-0.30 channel with 0.30 mm spacing, while the Triple-0.25 channel with 0.25 mm spacing had the lowest average flow velocity. The comparison reveals that within the three-inlet channels, the average flow velocity first decreased and then increased as the channel spacing increased from 0.20 mm to 0.30 mm.

Comparing the average flow velocities of microchannels with identical channel spacing, the Single-0.20 channel exhibits a higher average velocity than the Triple-0.20 channel, and the Single-0.25 channel demonstrates a higher average velocity than the Triple-0.25 channel. However, the Single-0.30 channel shows a lower average velocity than the Triple-0.30 channel.

The effectiveness of flow diversion can be compared based on the degree of fluctuation in flow velocity within each straight channel. Greater fluctuation indicates more pronounced velocity differences, resulting in poorer diversion performance. The uniformity parameter of flow velocity within straight channels characterizes the uniformity of the velocity distribution in microchannels. A smaller value for *Q_SSD_* indicates better flow distribution uniformity. After measuring flow velocities in straight channels using the PIV system, the following calculation yields the following:

(8) $$Q_{SSD}=\sqrt{\frac{\sum_{i=1}^{N}{\left(u_{i}-u_{\mathrm{avg}}\right)}^{2}}{N}}$$

In the formula, *u_i_* is the flow velocity within the straight channel and *u*_avg_ is the average value of flow velocities in all straight channels.

**Figure 17:**
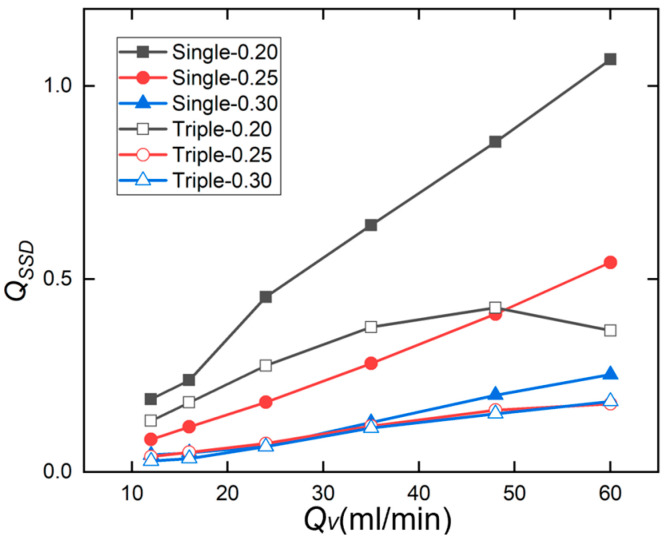
Variation in the non-uniformity parameter *Q_SSD_* for a straight microchannel with flow rate Qv.

Figure 17 shows the flow rate variation curves for different inlet configurations of straight microchannels, with channel spacings of 0.20 mm, 0.25 mm, and 0.30 mm. At a flow rate of 60 mL/min, within the field of view, the standard deviation of the flow velocity in the Triple-0.20 channel was 0.366, while that in the Single-0.20 channel under the same flow conditions was 1.069. This indicates that compared to the single-inlet straight microchannel with a channel spacing of 0.20 mm, the triple-inlet microchannel exhibits smoother flow velocity fluctuations and more uniform distribution. At a flow rate of 60 mL/min, the standard deviations of flow velocity for the Triple-0.25 and Triple-0.30 channels were 0.176 and 0.183, respectively. Both values were lower than the unevenness parameters of 0.543 and 0.248 for the Single-0.25 and Single-0.30 channels with the same channel spacing.

The comparison of these flow velocity standard deviations leads to the conclusion that the triple-inlet design achieves superior flow distribution compared to the single-inlet design.

### 3.2. Comparison of Maximum Temperatures for Heat Source Chips

**Figure 18:**
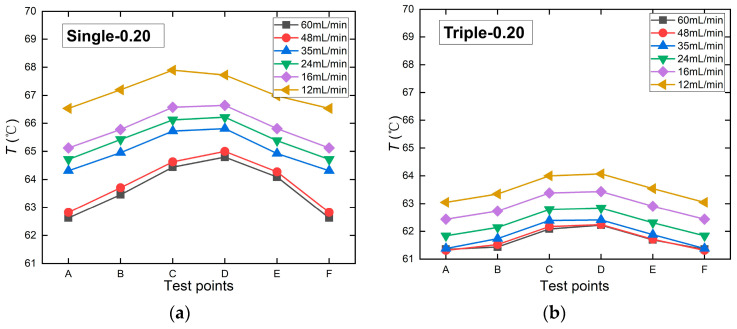
Temperature distribution map at various measurement points on a flat microchannel heat source chip with a channel spacing of 0.20 mm.

The thermal test chip is equipped with six temperature measurement points: A, B, C, D, E, and F. The test results indicate that the maximum temperature of the chip consistently occurs in the central region (specifically at measurement points C or D), and this trend holds for all microchannel structures. Therefore, by extracting the maximum temperatures from the central measurement points of each heat source chip under different flow rates, we obtained the maximum temperature variation curves with the flow rate for six flat microchannel structures, as shown in Figure 18.

**Figure 19:**
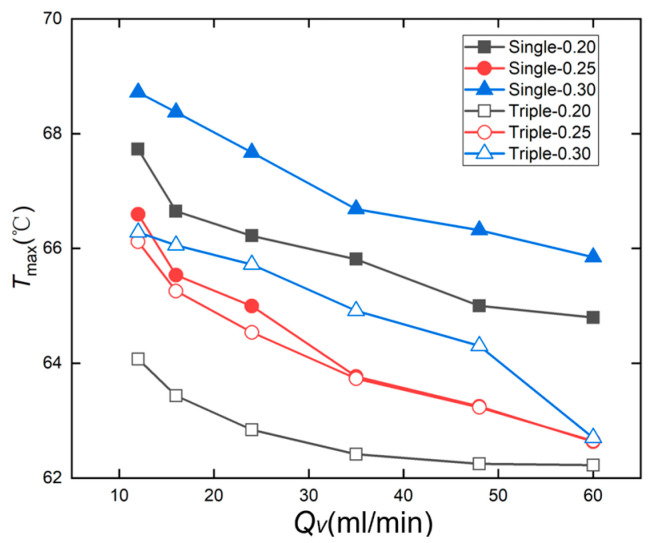
Variation in maximum temperature *T*_max_ with flow rate *Q_v_* in a straight microchannel chip.

The test results in Figure 19 show that the maximum chip temperature decreases as the flow rate increases. Comparing the maximum temperatures of chips with identical flow rates and identical channel spacings, but different inlet configurations, at a flow rate of 60 mL/min, the Single-0.20 chip reached 64.80 °C, while the Triple-0.20 chip reached 62.22 °C, indicating that the triple-inlet microchannel chip exhibited a lower maximum temperature than the single-inlet design. Similarly, the Triple-0.30 chip recorded 62.63 °C, lower than the Single-0.30’s 65.85 °C. The Single-0.25 and Triple-0.25 chips exhibited comparable maximum temperatures. Among these, the Triple-0.30 chip demonstrated the most effective heat dissipation, maintaining a maximum temperature of 62.22 °C. By comparing the variation patterns of the maximum temperature (*T*_max_) with the flow rate across different channel spacing and configuration designs, it is evident that *T*_max_ decreases with increasing flow rate for all configurations. Notably, the single-channel configuration consistently exhibits higher *T*_max_ than the three-channel design. This difference becomes more pronounced at smaller channel spacings (0.20 mm) and lower flow rates, indicating that the synergistic effect of multiple inlets and narrow spacing significantly enhances the chip’s heat dissipation performance.

**Figure 20:**
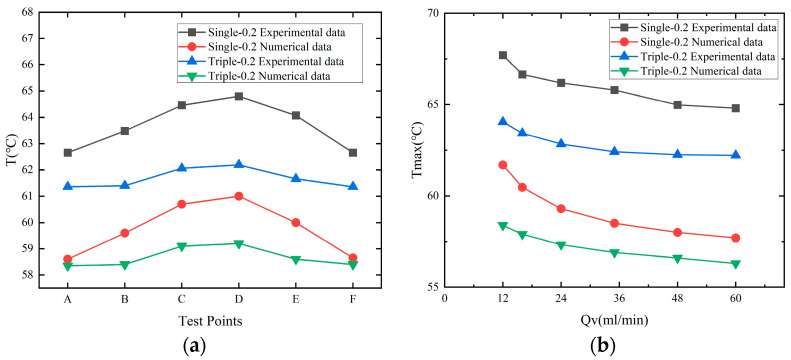
Comparison of experimental and simulated temperature distributions for a microfluidic channel with a flow rate of 60 mL/min and a channel spacing of 0.20 mm. (**a**) Temperature comparison at various measurement points on the heat source chip. (**b**) Variation in maximum temperature *T*_max_ with flow rate *Q_v_*.

A comparative analysis of experimental data and numerical simulation results under Single-0.2 and Triple-0.2 operating conditions, as shown in Figure 20, indicates that under varying flow rates (*Q_v_*) and across all test points (A–F), the temperature variation trends from numerical simulations closely align with experimental measurements. Whether for single-channel or three-channel microchannels, the simulation data accurately captures the overall pattern: the temperature decreases with the increasing flow rate and exhibits a rise followed by a fall along the test points. Furthermore, the numerical discrepancies between the two are minimal, validating the reliability of the established numerical model in predicting microchannel heat transfer characteristics. Meanwhile, the Triple-0.2 three-channel configuration consistently maintained lower temperatures than the Single-0.2 single-channel design, demonstrating superior thermal dissipation performance. Furthermore, the three-channel system exhibited reduced temperature fluctuations and more uniform distribution, further evidencing the advantages of a multi-channel design in enhancing heat exchange and improving temperature uniformity. These findings not only deepen our understanding of the heat transfer mechanisms within multi-channel microfluidic channels but also provide robust experimental and simulation support for the structural optimization and engineering applications of microfluidic heat dissipation systems.

### 3.3. Pressure Drop Comparison

**Figure 21:**
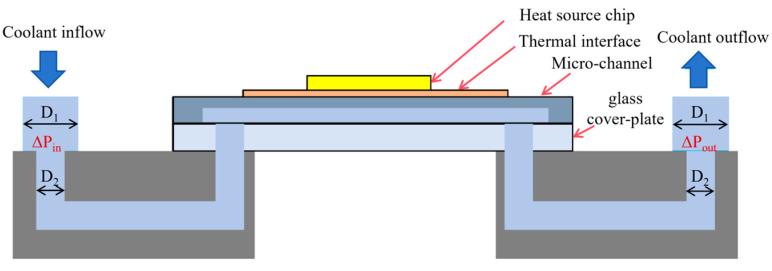
Schematic diagram of pressure drop loss.

Figure 21 illustrates the pressure drop loss. Coolant enters the fixture through a vertical downward flow path with an inner diameter D_1_ of 2.5 mm, then passes through a channel with an inner diameter D_2_ of 1 mm. After flowing through the flow-guiding structure, it enters the microchannel sample and exits the fixture via symmetrically arranged paths.

The pressure drop in this process primarily includes two components: the contraction pressure drop Δ*P_in_* caused by the sudden contraction at the inlet when the coolant enters the fixture from the connecting tubing, and the expansion pressure drop Δ*P_out_* caused by the sudden expansion at the outlet when the coolant exits the fixture. During testing, a pressure transducer measures the pressure drop at the inlet and outlet of the microchannel fixture, yielding the measured value Δ*P_meas_*. The microchannel pressure drop Δ*P_ch_* can be calculated using the following formula:

(9) $$∆P_{ch}=∆P_{meas}-∆P_{loss}$$

The pressure drop loss Δ*P_loss_* can be calculated using the following formula.

(10) $$∆P_{loss}=∆P_{in}+∆P_{out}$$

The pressure drop loss Δ*P_in_* caused by the sudden contraction at the inlet is shown in the formula.

(11) $$\Delta P_{in}=\rho_{f}\frac{{u_{1}}^{2}}{2}(1-(\frac{A_{2}}{A_{1}}{)}^{2}+\frac{1}{2}(1-\frac{A_{2}}{A_{1}}))$$

In the equation, *u*_1_ is the flow velocity after entering the contraction section, *A*_1_ is the cross-sectional area of the pipeline with inner diameter D_1_, and *A*_2_ is the cross-sectional area of the diversion structure with inner diameter D_2_.

Pressure drop loss Δ*P_out_* caused by sudden expansion at the outlet:

(12) $$\Delta P_{out}=\rho_{f}\frac{{u_{2}}^{2}}{2}(1-\frac{A_{2}}{A_{1}}{)}^{2}$$

In the equation, *u*_2_ is the flow velocity before the flow expansion section, *A*_1_ is the cross-sectional area of the pipeline with inner diameter D_1_, and *A*_2_ is the cross-sectional area of the flow diversion structure with inner diameter D_2_.

The calculated pressure drop loss Δ*P* of the microchannel adapter fixture at different flow rates is shown in Table 5.

**Figure 22:**
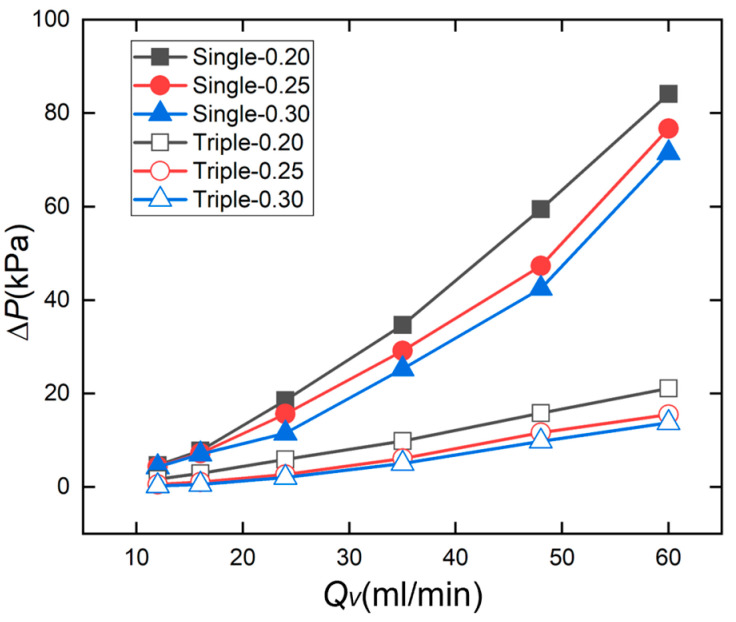
Pressure drop Δ*P* vs. volume flow rate *Q_v_* for straight microchannel.

Figure 22 shows the pressure drop versus the flow rate curve for a straight microchannel heat sink. The test results indicate that the pressure drop increases with the rising flow rate. Comparing the effect of channel spacing on pressure drop, for the single-inlet configuration at 60 mL/min flow rate, the pressure drops were 84.13 kPa for Single-0.20, 76.74 kPa for Single-0.25, and 71.51 kPa for Single-0.30. For the triple-inlet configuration at 60 mL/min flow rate, the pressure drops for Triple-0.20, Triple-0.25, and Triple-0.30 were 21.10 kPa, 15.54 kPa, and 13.75 kPa, respectively. As the channel spacing increased from 0.20 mm to 0.30 mm, the pressure drop decreased.

The pressure drop of all configurations increases approximately linearly with the rising flow rate. Among them, the single-inlet configuration consistently exhibits a significantly higher pressure drop increase rate and absolute value compared to the triple-inlet configuration. Simultaneously, within the same channel configuration, a smaller channel spacing results in a greater pressure drop. This indicates that increasing the number of channels effectively distributes the flow to reduce flow resistance, whereas a smaller channel spacing increases the flow resistance due to the reduced cross-sectional area of the channels. This result reveals the synergistic regulatory effect of channel configuration and channel spacing on flow resistance. In microfluidic chip design, a trade-off between the heat dissipation efficiency and pressure drop is necessary to achieve optimal performance.

### 3.4. Comprehensive Performance Evaluation of Microfluidic Structures

The performance evaluation criterion (PEC) was used to characterize the comprehensive performance of the microchannels [25], which allows for a holistic assessment of factors (e.g., improved heat transfer efficiency and system pressure drop) for different microchannels operating under the same flow rates. A PEC value exceeding one indicates superior heat transfer enhancement capabilities, relative to the reference channel. This metric is calculated using Equation (13):

(13) $$PEC=\frac{\left(Nu_{2}/Nu_{1}\right)}{\left(f_{2}/f_{1}\right)}$$

In the equation, *Nu*_1_ and *f*_1_ represent the Nusselt number and resistance factor of the reference flow channel, respectively, and *f* is calculated from Equation (14).

(14) $$f=\frac{2\Delta P}{\rho_{f}{u_{avg}}^{2}N_{i}}$$

In the formula, Δ*P* denotes the pressure drop across the flow channel and *N_i_* denotes the number of transverse rows within the flow channel.

Equation (13) transformed to compare the performance metrics σ of microchannels across different structures, as shown in Equation (15):

(15) $$\sigma =\frac{Nu}{f}$$

Comparing the performance metrics σ of various microchannel structures under a flow rate of 60 mL/min, the performance parameters for each straight microchannel are presented in Table 6. Among the straight microchannels, Straight-0.2-Triple demonstrated the most optimal overall performance.

## 4. Conclusions

This study first analyzed experimental data from PIV visualization experiments, extracted the average flow velocity in each straight channel, and compared the standard deviations of flow velocities across different flow paths to determine the superiority of the single-inlet versus triple-inlet configurations in terms of flow distribution. Subsequently, it compared the maximum surface temperatures and pressure drops across microchannels with varying channel spacings and inlet configurations. Finally, channel optimization was performed by evaluating the comprehensive performance metrics of each channel configuration. This process identified the optimal microchannel structure, exhibiting high heat transfer efficiency, low pressure drop, and uniform temperature distribution. The key conclusions are as follows:

1. The triple-inlet structure significantly improves the uniformity of the in-channel flow velocity distribution and eliminates the common “high in the middle, low on both sides” velocity pattern observed in single-inlet designs. PIV experiments demonstrate that the triple-inlet structure exhibits smaller velocity differences across channels and superior flow splitting efficiency. As the channel spacing decreases from 0.30 to 0.20, this splitting advantage becomes increasingly pronounced, thereby effectively enhancing coolant utilization.
2. Under identical flow conditions, the maximum temperature of chips in triple-inlet microchannels is lower than that in single-inlet microchannels with the same channel configuration, indicating superior heat transfer performance for the triple-inlet design. Under identical flow conditions, the pressure drop in triple-inlet channels is significantly lower than in single-inlet channels, ranging from 11.1% to 26.6% of the single-inlet value.

## Figures and Tables

**Table 1:** Microchannel sample geometry.

Number	Sample Code	Rib Diameter D (mm)	Channel Spacing W (mm)	Number of Channels N
1	Single-0.20	0.1	0.20	35
2	Single-0.25	0.1	0.25	28
3	Single-0.30	0.1	0.30	23
4	Triple-0.20	0.1	0.20	35
5	Triple-0.25	0.1	0.25	28
6	Triple-0.30	0.1	0.30	23

**Table 2:** Experimental and data reduction uncertainty.

Name	Uncertainty
Flow rate	±0.8%
Temperature of water	±0.67%
Temperature of heat source chip	±0.5%
Pressure	±0.5%

**Table 3:** Structural dimension parameters of the microchannel simulation model.

Name	Heat Source Chip	Thermal Interface Material	Microchannel Substrate	Fluid Domain	Glass Cover Plate
Length × Width × Height (mm)	2 × 6.6 × 0.4	6 × 7 × 0.1	10 × 10 × 0.5	8 × 7 × 0.25	10 × 10 × 0.5

**Table 4:** Relative error statistics.

Sample Code	Number of Selected Grid Cells (×10^6^)	*T*_max_ Relative Error (%)	Δ*P* Relative Error (%)
Single-0.20	3.075	0.020	4.627
Single-0.25	2.32	0.039	3.970
Single-0.30	2.226	0.039	4.308
Triple-0.20	3.468	0.18	3.947
Triple-0.25	4.312	0.164	2.470
Triple-0.30	2.579	0.235	3.276

**Table 5:** Pressure drop loss of microchannel fixture at different flow rates.

Volume flow *Q_v_* (mL/min)	60	48	35	24	16	12
Pressure drop loss Δ*P_loss_* (kPa)	1.81	1.16	0.62	0.29	0.13	0.07

**Table 6:** Performance parameters of each microchannel.

Straight Microchannel—Single Inlet	*σ*	Straight Microchannel—Triple Inlet	*σ*
Straight-0.2-Single	6.74	Straight-0.2-Triple	12.90
Straight-0.25-Single	2.21	Straight-0.25-Triple	4.87
Straight-0.3-Single	0.50	Straight-0.3-Triple	11.13

**Figure A1:**
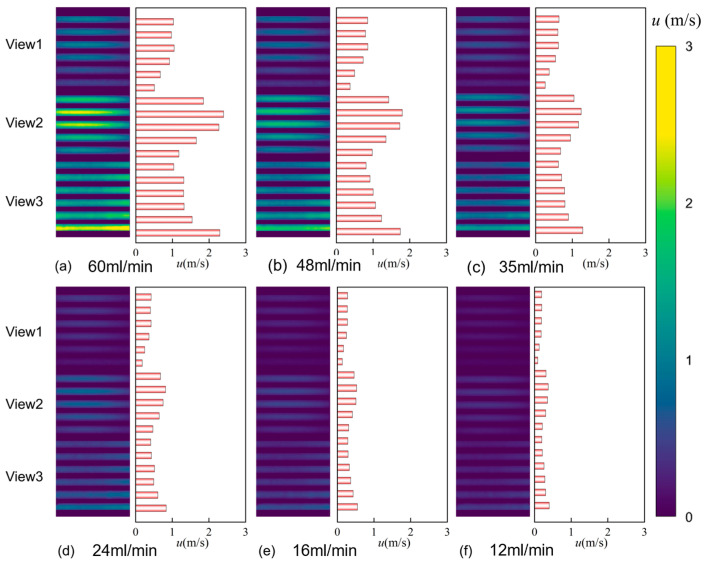
PIV velocity distribution maps of the Single-0.25 microchannel at different flow rates: (**a**) 60 mL/min, (**b**) 48 mL/min, (**c**) 35 mL/min, (**d**) 24 mL/min, (**e**) 16 mL/min, and (**f**) 12 mL/min.

**Figure A2:**
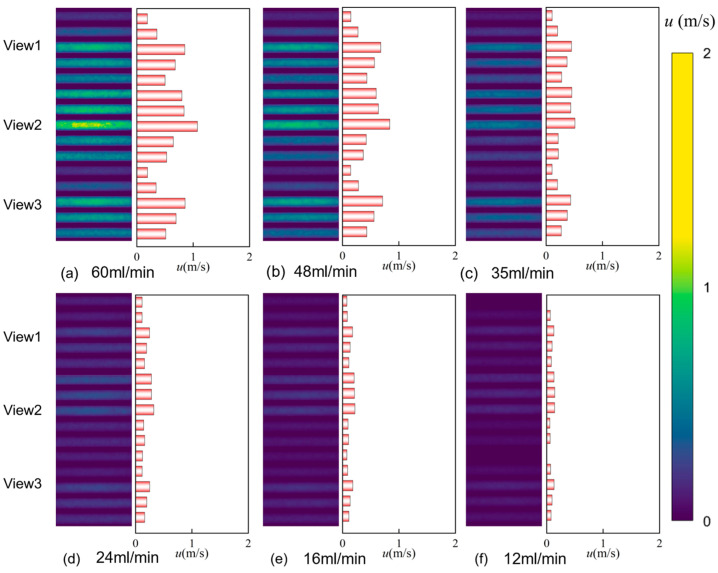
PIV velocity distribution maps of the Triple-0.25 microchannel at different flow rates: (**a**) 60 mL/min, (**b**) 48 mL/min, (**c**) 35 mL/min, (**d**) 24 mL/min, (**e**) 16 mL/min, and (**f**) 12 mL/min.

**Figure A3:**
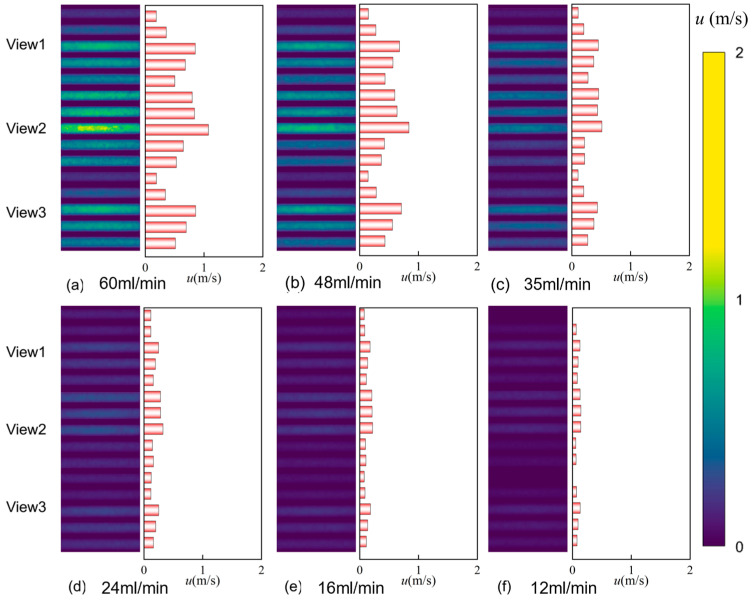
PIV velocity distribution maps of the Single-0.30 microchannel at different flow rates: (**a**) 60 mL/min, (**b**) 48 mL/min, (**c**) 35 mL/min, (**d**) 24 mL/min, (**e**) 16 mL/min, and (**f**) 12 mL/min.

**Figure A4:**
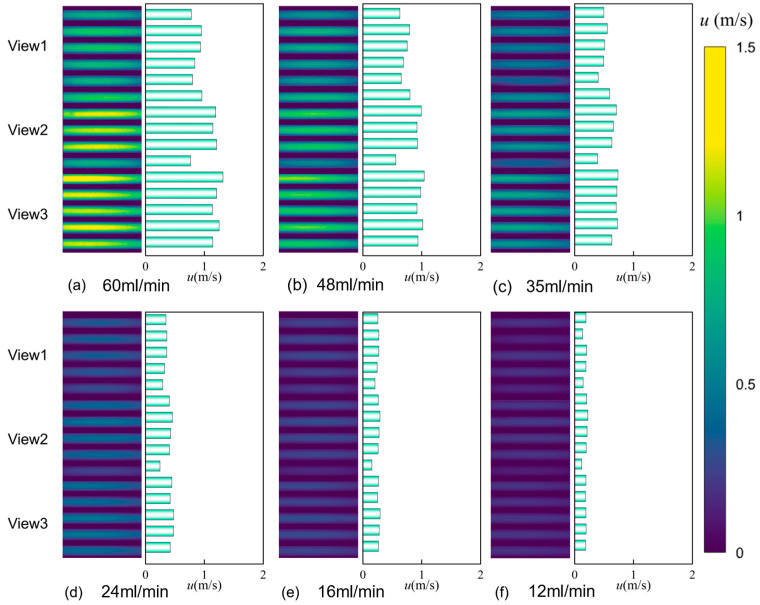
PIV velocity distribution maps of the Triple-0.30 microchannel at different flow rates: (**a**) 60 mL/min, (**b**) 48 mL/min, (**c**) 35 mL/min, (**d**) 24 mL/min, (**e**) 16 mL/min, and (**f**) 12 mL/min.

## Data Availability

The original contributions presented in this study are included in the article. Further inquiries can be directed to the corresponding author.
