# Correction: Xi et al. Influence of Inlet Splitter Structure on Flow and Heat Transfer Performance in Microchannel Heat Exchangers. *Micromachines* 2026, *17*, 275

**DOI:** 10.3390/mi17040443

**Published:** 2026-04-02

**Authors:** Yuanyuan Xi, Si Chen, Wenchao Tian, Xiong Xiao, Shuaike Li, Feiyang Li, Yifan Wang, Haojie Dang

**Affiliations:** 1 School of Electro-Mechanical Engineering, Xidian University, Xi’an 710071, China; 24041212451@stu.xidian.edu.cn (Y.X.); 24041111090@stu.xidian.edu.cn (F.L.); 24041212624@stu.xidian.edu.cn (Y.W.); d2100710345@outlook.com (H.D.); 2 The Fifth Electronics Research Institute of Ministry of Industry and Information Technology, Guangzhou 511370, China; 3 State Key Laboratory of Electromechanical Integrated Manufacturing of High-Performance Electronic Equipments, Xi’an 710071, China; 4 Pen-Tung Sah Institute of Micro-Nano Science and Technology, Xiamen University, Xiamen 361005, China; xiaoxiong@stu.xmu.edu.cn; 5 State Grid Electric Power Research Institute Co., Ltd. (NARI Group Co., Ltd.), Nanjing 211106, China; lishuaike@sgepri.sgcc.com.cn

Following publication, the authors made an administrative error during submission. The author order does not fully reflect the actual contributions of each author, and a correction is required. With this correction, the Editorial Office and the authors have made the following amendments to the published article [1] by changing the authorship order and adding Xiong Xiao as a new author:

This correction changes “Wenchao Tian ^1,2,^*, Yuanyuan Xi ^1,^*, Shuaike Li ^3^, Feiyang Li ^1^, Yifan Wang ^1^, Haojie Dang ^1^ and Si Chen ^4^” to “Yuanyuan Xi ^1^, Si Chen ^2,^*, Wenchao Tian ^1,3,^*, Xiong Xiao ^4^, Shuaike Li ^5^, Feiyang Li ^1^, Yifan Wang ^1^ and Haojie Dang ^1^”. The corresponding authors have also been updated from “wctian@xidian.edu.cn (W.T.); 24041212451@stu.xidian.edu.cn (Y.X.)” to “chensiceprei@yeah.net (S.C.); wctian@xidian.edu.cn (W.T.)”. Also, affiliation “Pen-Tung Sah Institute of Micro-Nano Science and Technology, Xiamen University, Xiamen 361005, China” has been added.

The “Author Contributions” section has been amended to reflect the complete attribution of all authors.

**Original text:**

Conceptualization, Methodology, Software., W.T.; Writing the draft, Validation, Y.X.; Project Administration, Formal Analysis, S.L., H.D. and F.L.; Project Administration, Y.W. and S.C. All authors have read and agreed to the published version of the manuscript.

**Corrected text:**

Sample design and fabrication, X.X.; Conceptualization, Methodology, Software, S.C. and W.T.; Writing the draft, Validation, Y.X.; Project Administration, Formal Analysis, S.L., H.D. and F.L.; Project Administration, Y.W. and S.C. All authors have read and agreed to the published version of the manuscript.

The information regarding the material provider was not fully annotated. We hereby provide the following supplementary clarification:

**Acknowledgments:**

The sample design and fabrication were carried out by the research team led by Shenglin Ma at Xiamen University. We extend our sincere gratitude to this team. All related work strictly adheres to the principle of respecting intellectual property rights. This supplementary acknowledgement and clarification are provided.

The authors state that the scientific conclusions are unaffected. This correction was approved by the Academic Editor. The original publication has also been updated.
